# Inhibitory Effect of *Lactobacillus Paracasei* CMU-Pb-L5 In a Subcutaneous Transplanted Tumor Model of Colorectal Cancer

**DOI:** 10.7150/ijms.99646

**Published:** 2024-09-30

**Authors:** Xiaodan Chang, Shaobing Zhang, Cong Li, Hailiang Zhang, Weiqing Yang, Weijian Zhang, Ziyu Ye, Yanfang Liang, Xianxiu Qiu, Jincheng Zeng

**Affiliations:** 1 Department of Neonatology, The Second Central Hospital of Baoding, Baoding 071051, China.; 2 Dongguan Key Laboratory of Medical Bioactive Molecular Developmental and Translational Research, Guangdong Provincial Key Laboratory of Medical Immunology and Molecular Diagnostics, Guangdong Medical University, Dongguan 523808, China.; 3 Dongguan Key Laboratory of Molecular Immunopathology, Department of Pathology, Binhaiwan Central Hospital of Dongguan, Dongguan 523000, China.; 4 Community health service center of Dongguan Dalang Town, Dongguan 523000, China.; 5 Xinghai Institute of Cell, Guangdong Xianhua Institute for Medical Research, Dongguan 523808, China.; 6 Dongguan Key Laboratory of Metabolic Immunology and Oral Disease, Department of Stomatology, Dongguan Maternal and Child Health Care Hospital, Dongguan 523000, China.

**Keywords:** *Lactobacillus paracasei*, colorectal cancer, polyamine metabolism, apoptosis

## Abstract

*Lactobacillus paracasei* (*L.p*) is a prevalent probiotic strain within the *Lactobacillus* genus, which has robust intestinal colonization capabilities. Previous studies have demonstrated the anticancer properties of *L.p* both *in vivo* and *in vitro*. However, the mechanisms underlying its anticancer activity *in vivo* remain unclear. This study established a subcutaneous transplanted tumor model of colorectal cancer (CRC) in mice to investigate the impact of *L.p* CMU-Pb-L5. Various parameters including tumor volume, tumor weight, histological alterations in tumor tissue, levels of polyamines and immune-related cytokines in serum, as well as the expression of polyamine metabolism-related and apoptosis-related proteins were evaluated. The results suggested that *L.p* CMU-Pb-L5 exhibited inhibitory effects on tumor cell proliferation, promotion of tumor cell apoptosis, reduction in polyamine levels, and enhancement of the immune response in CRC mice. To sum up, these results suggested that *L.p* CMU-Pb-L5 holds promise for potential clinical applications in the treatment of CRC.

## 1. Introduction

Colorectal cancer (CRC) is a prevalent malignancy globally, ranking third in incidence and second in mortality among all cancers in 2020^1^. Alarmingly, its incidence is rising, particularly among younger individuals^2^. Various factors contribute to CRC, including chronic inflammation, familial predisposition, poor dietary habits, lifestyle choices, and dysbiosis of the gut microbiota^3^. While genetic factors are more significant in hereditary CRC cases, environmental factors play a major role in sporadic CRC, with increasing attention on the role of the microbiota in cancer biology^4^.

Polyamine metabolites are closely associated with oncogenes and various carcinogenic signaling pathways^5^, such as the PI3K/Akt^6^, Hedgehog^7^, RAS/RAF/MEK^8^, and MYC signaling pathway^9^. Tumor cells require high levels of polyamines like putrescine (PUT), spermidine (SPD), and spermine (SPM) for proliferation, and enzymes involved in polyamine metabolism (such as ODC, SMOX, SRM) are potential targets for cancer therapy^10^, ^11^. Gastrointestinal polyamines, sourced from diet and intestinal flora, contribute to tumor growth, especially when there's an imbalance in the gut microbiota. Pathogens in the gut can produce polyamines supporting tumor growth^12^, ^13^.

In many cancers, including CRC, polyamine levels are elevated^11^. Polyamine metabolism plays a crucial role in apoptosis and proliferation of cancer cells. Polyamines can also promote tumor migration and invasion by facilitating the binding of transcription factors to specific response elements^14^.

For instance, serum polyamines are significantly elevated in prostate cancer patients^15^ indicating their role in maintaining cell proliferation and the possibility of targeting polyamine metabolism as a therapeutic strategy. The findings emphasize the importance of polyamines and their metabolism in CRC development and suggest potential avenues for therapeutic intervention^16^. Studies dating back to the 1980s^17^ have shown significantly increased polyamine levels in CRC tissues, blood, and urine samples, suggesting their potential as markers for understanding CRC carcinogenesis^18^, ^19^.

CRC has been linked to an imbalance in intestinal flora, establishing it as a microbiome-related disease^20^. Probiotics, which are beneficial microorganisms that colonize the host intestine and regulate its flora, have shown promise as adjuvant treatments for CRC^21^. Clinical studies have demonstrated that preoperative supplementation of probiotics in CRC patients can reduce serum endotoxin and C-reactive protein levels while increasing peripheral blood immunoglobulin content^22^. *Lactobacillus paracasei* (*L.p*) is known for its immunomodulatory effects, ability to regulate flora balance, inhibit pathogenic bacteria, and exhibit anti-tumor activity^23^, ^24^. Studies have shown that *L.p* has significant anti-cancer effects both *in vivo* and *in vitro*. For example, *L.p* K5 has been shown to adhere strongly to colon cancer cells, inhibit their proliferation, and induce apoptosis through the regulation of Bcl-2 family proteins^25^. Similarly, *L.p* X12 was found to effectively inhibit tumor growth in CRC rats induced by carcinogenic chemicals^26^.

However, the precise mechanism of *L.p*'s anti-cancer activity *in vivo* has not been fully elucidated. *L.p* CMU-Pb-L5, which has demonstrated good tolerance to gastric and intestinal fluids as well as safety, has been shown in previous studies to regulate the balance of pro-inflammatory and anti-inflammatory factors in colitis models, thereby alleviating colitis symptoms and pathological damage^27^. This study aims to establish a mouse model of CRC and investigate how *L.p* CMU-Pb-L5 regulates polyamine metabolism to inhibit CRC. By focusing on this aspect, the study seeks to provide a better understanding of the mechanisms underlying *L.p* CMU-Pb-L5's anti-cancer activity in mice, offering a foundation for its potential clinical application.

## 2. Materials and Methods

### 2.1 *Lactobacillus Paracasei* strain

*Lactobacillus Paracasei* strain CMU-Pb-L5 was kindly gifted by Guangdong Xinghai Biotechnology Co. Ltd. CMU-Pb-L5 was grown in MRS Broth (Oxoid).

### 2.2 Cell culture

Human colorectal cancer cell line SW1116 and RKO, murine colon carcinoma cell line CT26 were purchased from the Cell Bank Type Culture Collection of the Chinese Academy of Sciences (Shanghai, China). SW1116, RKO and CT26 cells were cultured in DMEM (Gibco) containing 1% penicillin/streptomycin (Gibco) and 10% fetal bovine serum (Biological Industries) at 37℃ in a 5% CO_2_ incubator.

### 2.3 Animals

Balb/c mice of 8-week-old were obtained from the Experimental Animal Center of Southern Medical University. All animal protocols were in accordance with ARRIVE guidelines and approved by the laboratory animal ethical committee of Guangdong Medical University in compliance with the National Guidelines for the Care and Use of Animals. Mice were housed in plastic cages (five animals per cage) in the animal facilities at 23 °C ± 2 °C and 40%-60% humidity with a 12-h dark/ light cycle and fed with a standard laboratory diet.

### 2.4 Cell viability assay

To examine the effect of CMU-Pb-L5 on colorectal cancer cell viability, the cells were seeded (5×10^3^ cells/well) in 96-well plates with DMEM containing 10% FBS. After 24 h, the cells were supplemented with fresh medium and treated with CMU-Pb-L5 (0, 1.25×10^6^, 2.5×10^6^ and 5×10^6^ CFU/mL) at different multiplicity of infection (MOI, 0, 50, 100, and 200) for 24 hours. Subsequently, the medium was aspirated and replaced with 100 μL of a 10% working solution of CCK-8 (Dojindo), followed by incubation for 2 hours. Finally, the absorbance was measured at 450 nm using a microplate reader (Thermo Fisher). All the assays performed in triplicate.

### 2.5 Flow cytometric analysis of apoptotic cells

RKO and SW1116 cells were inoculated into a 6-well plate at 2×10^5^ cells per well and incubated overnight. *L.p* CMU-Pb-L5 was co-cultured with SW1116 and RKO cells at an MOI of 10 for 24 hours. Apoptotic cells were detected using Annexin V, FITC apoptosis detection kit (Dojindo) according to the manufacturer's protocols and the stained cells were measured by flow cytometric analysis (BD FACSCalibur). Data were analyzed by FlowJo.V10 software. All the tests performed in triplicate.

### 2.6 Mouse model of colorectal cancer

Eighteen mice were randomly divided into three groups: control group, CMU-Pb-L5 group, and 5-fluorouracil group (5-Fu group), with six mice in each group. Mice in CMU-Pb-L5 group were given 200 μL of CMU-Pb-L5 (1×10^9^ CFU/mL) daily by gavage, while mice in control group and 5-Fu group received an equal volume of PBS solution.

Two weeks after CMU-Pb-L5 or PBS intervention, 5×10^5^ CT26 cells in 100 μL PBS were injected into the right armpit of all mice. Three days after armpit injection, mice in CMU-Pb-L5 group were given 200 μL of CMU-Pb-L5 (1×10^9^ CFU/mL) daily for 20 consecutive days by gavage, while mice in 5-Fu group were intraperitoneally administered with 5-Fu at 25 mg/kg every 3 days, mice in control group were given 200 μL of PBS daily for 20 consecutive days by gavage. All mice were weighed every 3 days. The long and short diameters of the tumors were measured with vernier calipers every 3 days. The tumor volume was calculated using the formula: volume = 1/2× width^2^× length. Twenty-three days after armpit injection, mice were sacrificed, blood, tumor tissues and organs were collected and prepared for further analysis. To determine the organ index of the liver and spleen in mice, use the following formula: organ index = M1/M2 [M1 is organ mass(g), M2 is weight mass (g)].

### 2.7 Polyamine content analysis by high performance liquid chromatography

The concentrations of putrescine, spermine, and spermidine in mouse serum were analyzed using high performance liquid chromatography (HPLC). Briefly, 50 μL of serum samples were added to 250 μL of 5% trichloroacetic acid, and agitated for 1 hour. Following centrifugation at 12,000 rpm and 4 °C for 10 minutes, 100 μL of the supernatant was collected for derivatization. Subsequently, the samples were derivatized with dansyl chloride, and analyzed by HPLC system (Waters e2695). Chromatographic conditions: mobile phase: A phase was ultrapure water, B phase was acetonitrile, gradient elution; detector: fluorescence detector; excitation wavelength is 340 nm, emission wavelength is 450 nm; flow rate: 0.8 mL/min; column temperature: 35 °C. All the assays performed in triplicate.

### 2.8 Cytokines analysis

The levels of IFN-γ, IL-2, IL-4, IL-10, IL-6, TNF-α, IL-1β, and IL-17A were analyzed by using Bio-Plex Pro Mouse Cytokine Assay kit (Bio-Rad) following manufacturer's instruction. The amounts of cytokines in serum (pg/mL) were measured on Bioplex 200 system and the obtained data were subjected to analysis by Bio-Plex Manager software version 6.0. All the tests performed in triplicate.

### 2.9 Histology and immunohistochemical analysis

Tumor tissues were fixed in 4% paraformaldehyde overnight at 4 °C and embedded in paraffin. For histological study, paraffin-embedded sections (4 μm) were stained with hematoxylin and eosin (H&E). For immunohistochemical analysis, the sections were deparaffinized and rehydrated. Following blocking with 2.5% goat serum in PBS, the sections were incubated overnight at 4 °C with primary antibodies: anti-Ki67, anti-SMOX, anti-ODC1 (Proteintech). Subsequently, the sections were detected using the universal immunoperoxidase ABC kit, followed by counterstaining with hematoxylin. Images were captured under a light microscope (Olympus) and semi-quantitative analysis was carried out using ImageJ software.

### 2.10 Western blot

The tumor tissues were lysed in RIPA lysis buffer (Beyotime), then ultrasonicated and incubated on ice. The lysates were centrifuged at 12,000 rpm for 15 min. Total protein concentration was detected by BCA protein assay kit (Beyotime). 50 μg of proteins from each sample were separated on SDS-PAGE gels and transferred onto 0.22 μm PVDF membranes (Millipore). The membranes were incubated at 4 °C overnight with primary antibodies: anti-SRM, anti-SMOX, anti-ODC1, anti-β-actin (Proteintech), anti-cMyc, anti-Bax, anti-Bcl-2, anti-Cleaved Caspase3, and anti-Caspase3 (Cell Signaling Technology). Subsequently, the membranes were incubated with HRP-conjugated secondary antibody (Beyotime). Finally, the protein bands on the membranes were visualized with enhanced chemiluminescence detection reagent (Millipore). The integrated density of the bands was quantitatively analyzed by using ImageJ software, the band of β-actin was used as an internal loading control. All the tests performed in triplicate.

### 2.11 Statistical Analysis

The measurement data were presented as mean ± SEM (standard error of the mean). The differences between two groups were analyzed using Student's *t*-test by GraphPad Prism 9.5 software. A *P*-value of < 0.05 was considered statistically significant.

## 3. Results

### 3.1 *L.p* CMU-Pb-L5 inhibited cell proliferation and induced cell apoptosis in a concentration-dependent manner in colorectal cancer cells

**Figure 1:**
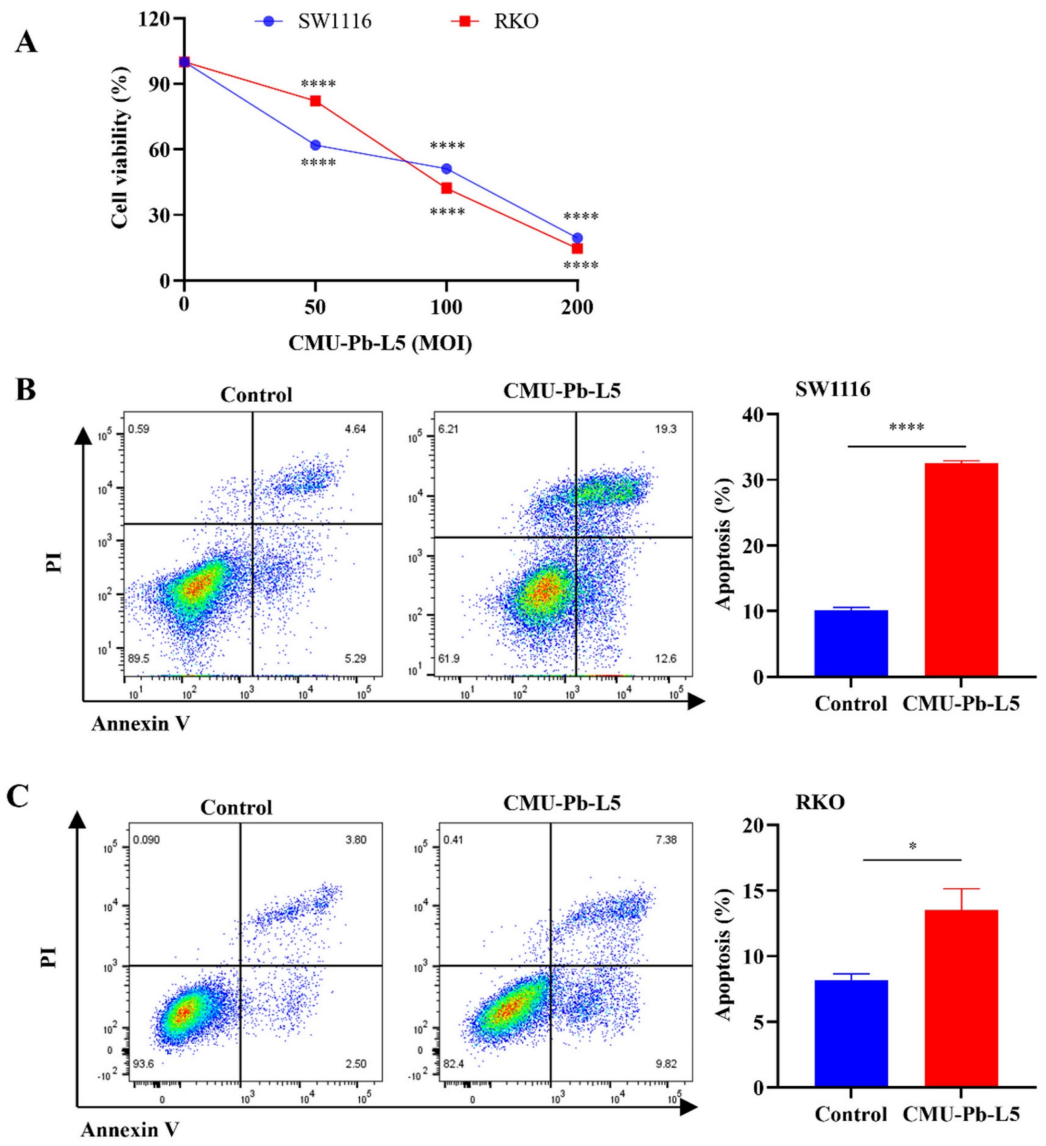
The impact of *Lactobacillus paracasei (L.p)* CMU-Pb-L5 on the proliferation and apoptosis of CRC cells *in vitro*. (**A**) Effects of *L.p* CMU-Pb-L5 on proliferation of CRC cells. ^****^, *P* < 0.0001, vs. uninfected group (MOI=0). (**B**) Effects of *L.p* CMU-Pb-L5 on apoptosis of SW1116 cells. ^****^, *P* < 0.0001. (**C**) Effects of *L.p* CMU-Pb-L5 on apoptosis of RKO cells. ^*^, *P* < 0.05.

The study investigated the impact of *Lactobacillus paracasei* (*L.p*) CMU-Pb-L5 on the proliferation and apoptosis of colorectal cancer (CRC) cells *in vitro*. Using the CCK-8 method, it was found that *L.p* CMU-Pb-L5 inhibited the proliferation of CRC cells SW1116 and RKO in a dose-dependent manner, with a more pronounced effect as the multiplicity of infection (MOI) increased (Figure 1A). Furthermore, flow cytometry analysis revealed that co-culturing CRC cells with *L.p* CMU-Pb-L5 at an MOI of 10 for 24 hours led to the promotion of apoptosis in SW1116 and RKO cells (Figure 1B, C). These results suggest that *L.p* CMU-Pb-L5 not only inhibits the proliferation but also promotes the apoptosis of CRC cells, indicating its potential as a therapeutic agent for CRC.

### 3.2 *L.p* CMU-Pb-L5 inhibited tumor growth in xenograft mice

**Figure 2:**
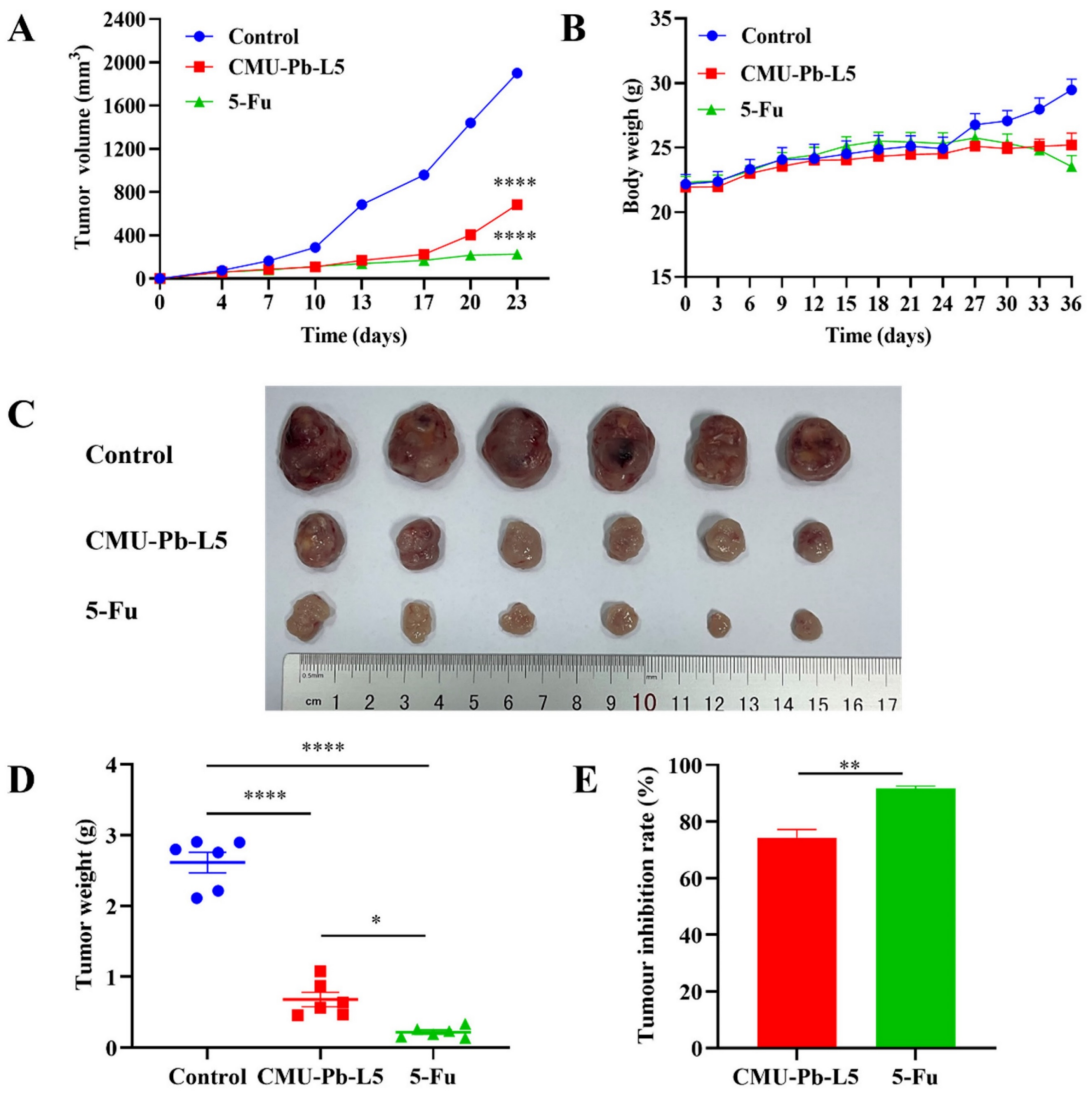
Effect of *L.p* CMU-Pb-L5 on tumor tissue growth in mice. (**A**) Growth curve of mouse tumor tissue. (**B**) Changes in body weight of mice. (**C**) Physical image of tumor tissue. (**D**) Mice tumor tissue weight. (**E**) Tumor inhibition rate. *, *P* < 0.05; **, *P* < 0.01; ****, *P* < 0.0001.

To further investigate the effect of *L.p* CMU-Pb-L5 on CRC, we established a mouse model of CT26 CRC. Over the initial one-week period from tumor cell inoculation to the first detection of solid tumors, control mice exhibited faster tumor growth (mean tumor volume increased by 211.57 mm^3^), while those in CMU-Pb-L5 group and the 5-Fu group showed slower growth. Ten days post-tumor cell inoculation, compared to control group, tumor tissue growth rates in CMU-Pb-L5 group and the 5-Fu group began to level off (Figure 2A). Throughout the modeling period to the experiment's end, the weight of mice in each group was monitored. It was observed that the weight of mice in control group and CMU-Pb-L5 group increased, while that of mice in the 5-Fu group showed a decreasing trend in the later period (Figure 2B), the difference was not statistically significant (*P* > 0.05).

After 23 days of tumor cell inoculation, the mice in each group were dissected, and the excised tumors were sequenced and photographed (Figure 2C). The mean tumor volume of mice in each group was as follows: (1901 ± 41.45) mm^3^ in control group, (685 ± 36.82) mm^3^ in CMU-Pb-L5 group, and (226 ± 9.08) mm^3^ in 5-Fu group. Compared with control group, the 5-Fu group had the smallest tumor volume, with a statistically significant difference (*P* < 0.0001), indicating the best tumor suppression effect. The tumor volume of CMU-Pb-L5 group was significantly lower than that of control group (*P* < 0.0001), indicating a better anti-tumor effect. Statistical analysis of the weight of excised solid tumors (Figure 2D) showed that the average tumor weight of CMU-Pb-L5 group was (0.6802 ± 0.1010) g, significantly lower than that of control group (2.614 ± 0.1452) g (*P* < 0.0001). The mean tumor weight of the 5-Fu group was (0.2197 ± 0.0304) g, also significantly lower than the other two groups (*P* < 0.0001). The tumor inhibition rates of each group were (91.47 ± 1.18) % in the 5-Fu group and (73.58 ± 3.93) % in CMU-Pb-L5 group (Figure 2E).

### 3.3 *L.p* CMU-Pb-L5 lowered the spleen and liver of mice with CRC

**Figure 3:**
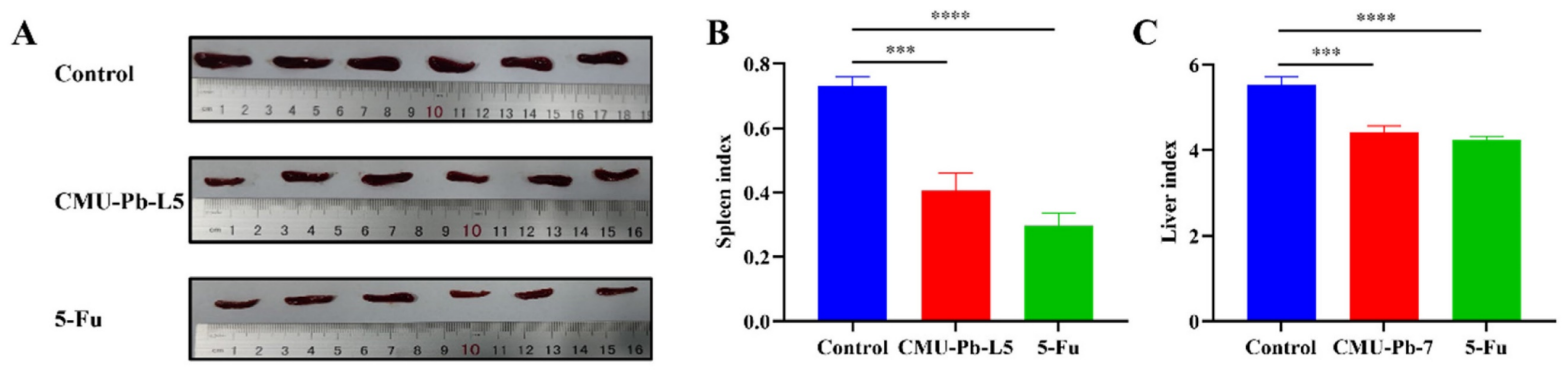
Effects of *L.p* CMU-Pb-L5 on spleen and liver in mice. (**A**) Images of excised spleens from mice. (**B**) Mouse spleen index. (**C**) Mouse liver index. ***, *P* < 0.001; ****,* P* < 0.0001.

After 23 days of tumor cell inoculation, the mice in each group were dissected, and their tissue organs were examined. Compared with control group, the spleens of mice treated with the *L.p* CMU-Pb-L5 strain or with intraperitoneal injection of the positive drug 5-Fu were both visibly decreased (Figure 3A). Both the spleen index and liver index of mice significantly decreased after *L.p* CMU-Pb-L5 administration as compared with the control group (Figure 3B, C).

### 3.4 *L.p* CMU-Pb-L5 affected tumor histomorphology in mice

**Figure 4:**
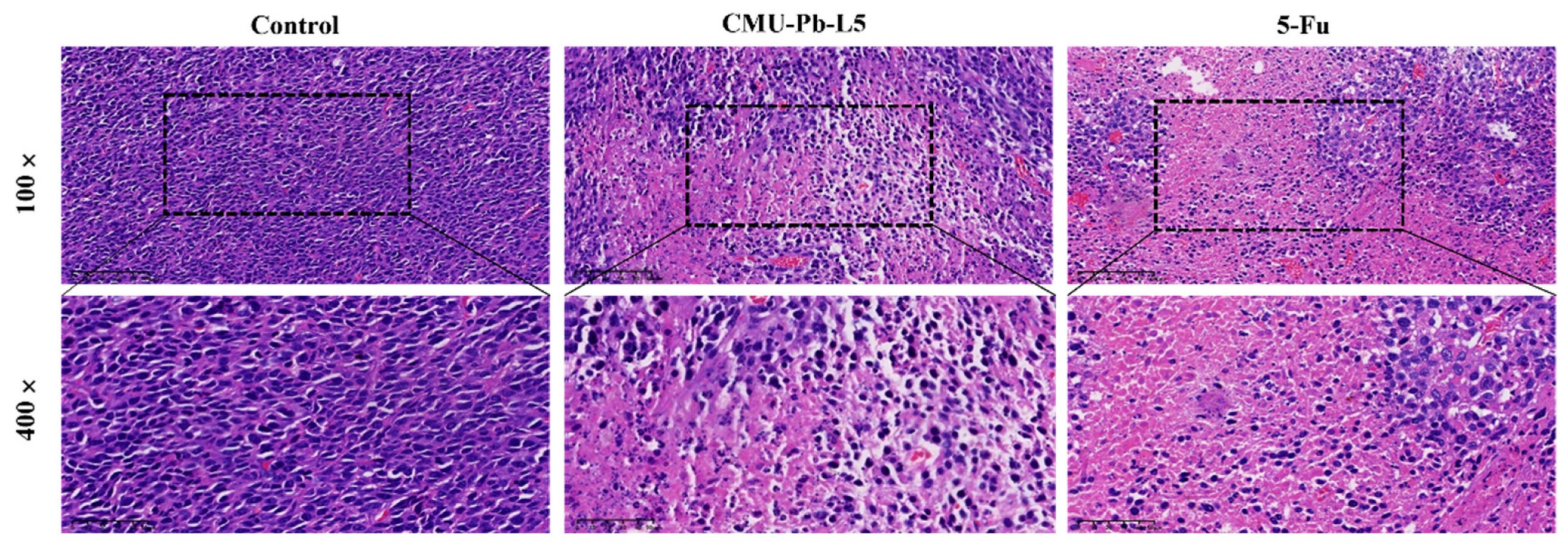
Effect of *L.p* CMU-Pb-L5 on tumor histomorphology in mice. H&E staining of paraffin-embedded sections of mice CRC tumor tissues.

Histological examination was conducted on the pathological sections of mouse tumor tissue in each group using hematoxylin and eosin (HE) staining. In control group, tumor cells were tightly arranged, exhibiting elliptical shapes with large, deeply stained nuclei, and sparse interstitial cells. In contrast, the tumor tissue of mice in CMU-Pb-L5 group and the positive drug 5-Fu group showed significantly reduced tumor cell density. These tissues displayed large, pink-stained areas, loosely arranged cells, and evidence of nucleolysis, nuclear fragmentation, and dissolution. Additionally, inflammatory cell infiltration and varying degrees of necrotic areas were observed compared to control group (Figure 4).

### 3.5 *L.p* CMU-Pb-L5 suppressed the expression of tumor-related protein in mice

**Figure 5:**
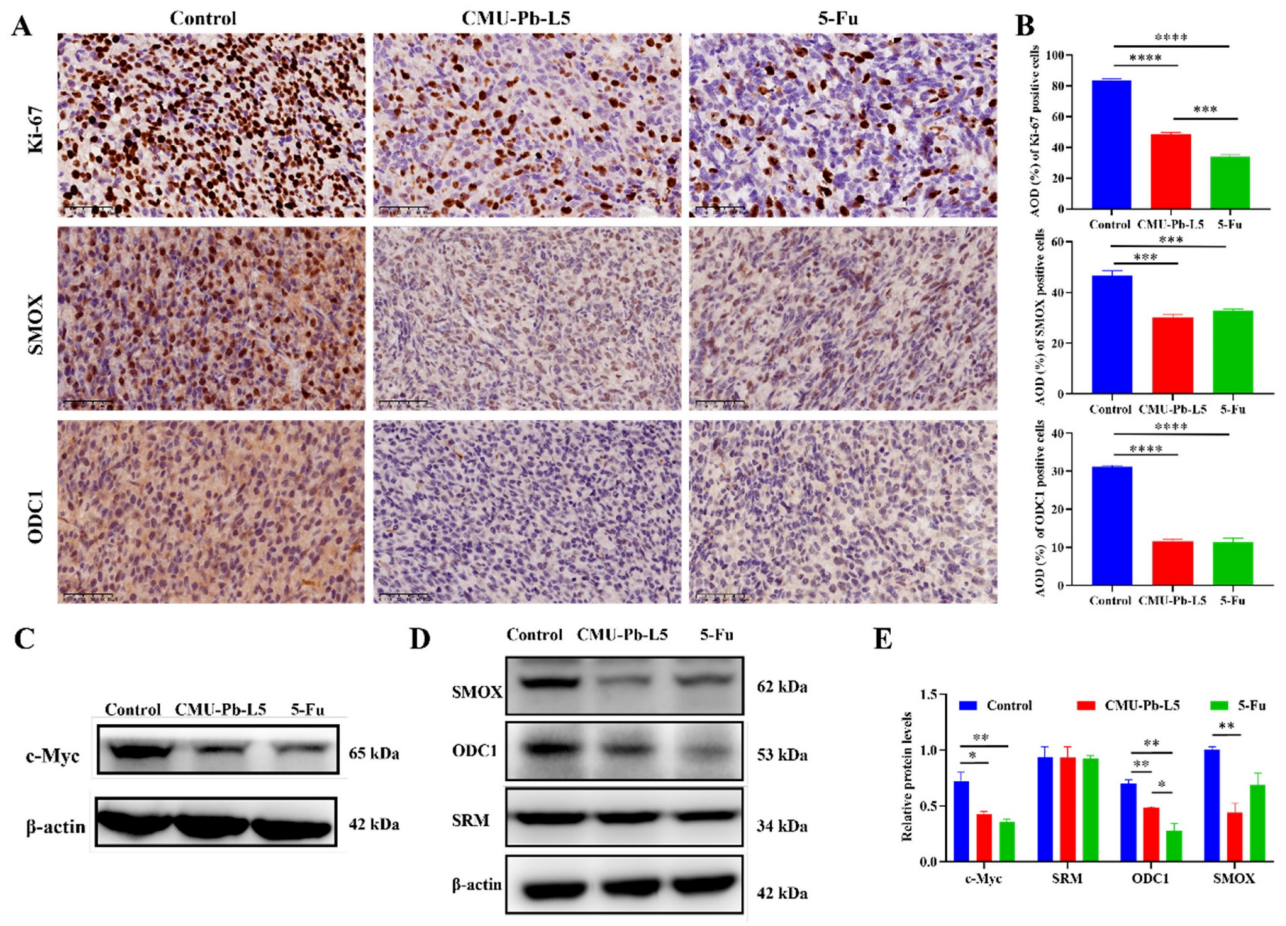
*L.p* CMU-Pb-L5 decreased the expression of tumor-related protein in mice. (**A**) Representative images for IHC analysis on the expression of Ki-67, SMOX and ODC1 in mice tumor tissues (400 ×). (**B**) Semi-quantitative analysis of IHC staining by ImageJ software. (**C**) The expression of c-Myc in mice tumor tissues was detected by Western blot. (**D**) The expression of polyamine metabolism-related proteins (SRM, ODC1, SMOX) in mice tumor tissues were detected by Western blot. (**E**) Representative semi-quantification of protein band densities normalized to β-actin from the Western blot analysis. *, *P* < 0.05; **, *P* < 0.01; ***, *P* < 0.001; ****, *P* < 0.0001.

Ki-67 is commonly used to assess cellular proliferative activity, and its protein expression is closely associated with tumor differentiation, invasion, metastasis, and prognosis^28^. To investigate the impact of *L.p* CMU-Pb-L5 on the proliferation of mouse tumor cells, the expression of Ki-67 protein in tumor tissues of mice in each group was examined. In control group, the number of Ki-67 positive cells was highest, with a relatively dense distribution. However, compared to control group, the number of Ki-67 positive cells decreased to some extent in both CMU-Pb-L5 group and 5-Fu group (Figure 5A). Semi-quantitative analysis of Ki-67 protein expression demonstrated a significant reduction after *L.p* CMU-Pb-L5 intervention compared to control group (*P* < 0.0001) (Figure 5B).

The oncogene c-Myc is often overexpressed in CRC, and its related signaling pathway is widely activated, promoting tumor initiation and progression^29^. To assess the effect of *L.p* CMU-Pb-L5 on c-Myc protein expression, we analyzed c-Myc protein levels in tumor tissues of mice in each group. Western blot results revealed high expression of c-Myc in control group, while its expression significantly decreased after *L.p* CMU-Pb-L5 intervention (*P* < 0.05) (Figure 5C).

To investigate the effect of *L.p* CMU-Pb-L5 on the expression of polyamine metabolism-related proteins in tumor tissues. Immunohistochemistry was utilized to assess the expression of SMOX and ODC1 in tumor tissues of mice in each group. The results demonstrated varying degrees of expression of SMOX and ODC1 in the tumor tissues of all groups (Figure 5A). Additionally, semi-quantitative analysis of positive cells in the tumor tissues revealed that the expression of SMOX and ODC1 in CMU-Pb-L5 group and 5-Fu group was significantly lower than that in control group (*P* < 0.001) (Figure 5B). Western blot results similarly indicated that the expression levels of ODC1 and SMOX proteins in tumor tissues following *L.p* CMU-Pb-L5 intervention were significantly decreased compared with control group (*P* < 0.01) (Figure 5D).

### 3.6 *L.p* CMU-Pb-L5 decreased serum polyamine in mice

**Figure 6:**
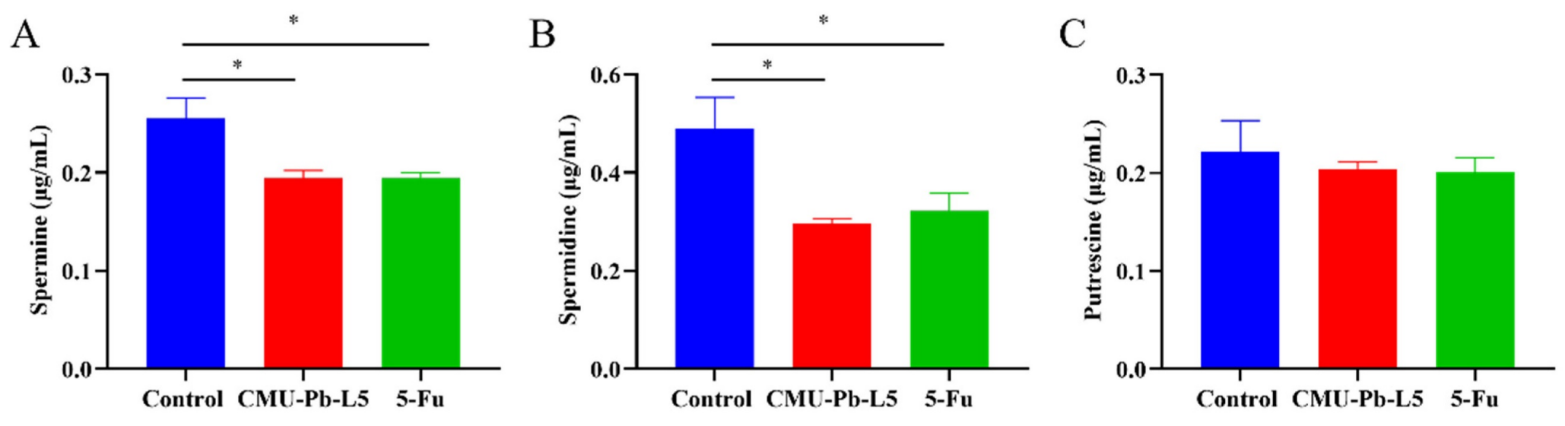
Effects of *L.p* CMU-Pb-L5 on serum polyamine content. The contents of spermine (**A**), spermidine (**B**), and putrescine (**C**) in mice serum were detected by HPLC. *, *P* < 0.05.

The polyamine metabolism in cancer patients often exhibits abnormalities, leading to significantly increased polyamine levels in the body. This elevated polyamine content provides favorable conditions for tumor metastasis and development^30^. Hence, we conducted further analysis to measure the serum polyamine levels in each group of mice using high performance liquid chromatography (HPLC). The results revealed that compared to control group, the levels of SPD (Figure 6B) and SPM (Figure 6A) in CRC mice significantly decreased following intervention with *L.p* CMU-Pb-L5 (*P* < 0.05) (Figure 6).

### 3.7 *L.p* CMU-Pb-L5 had the function of regulating immune-related cytokines

**Figure 7:**
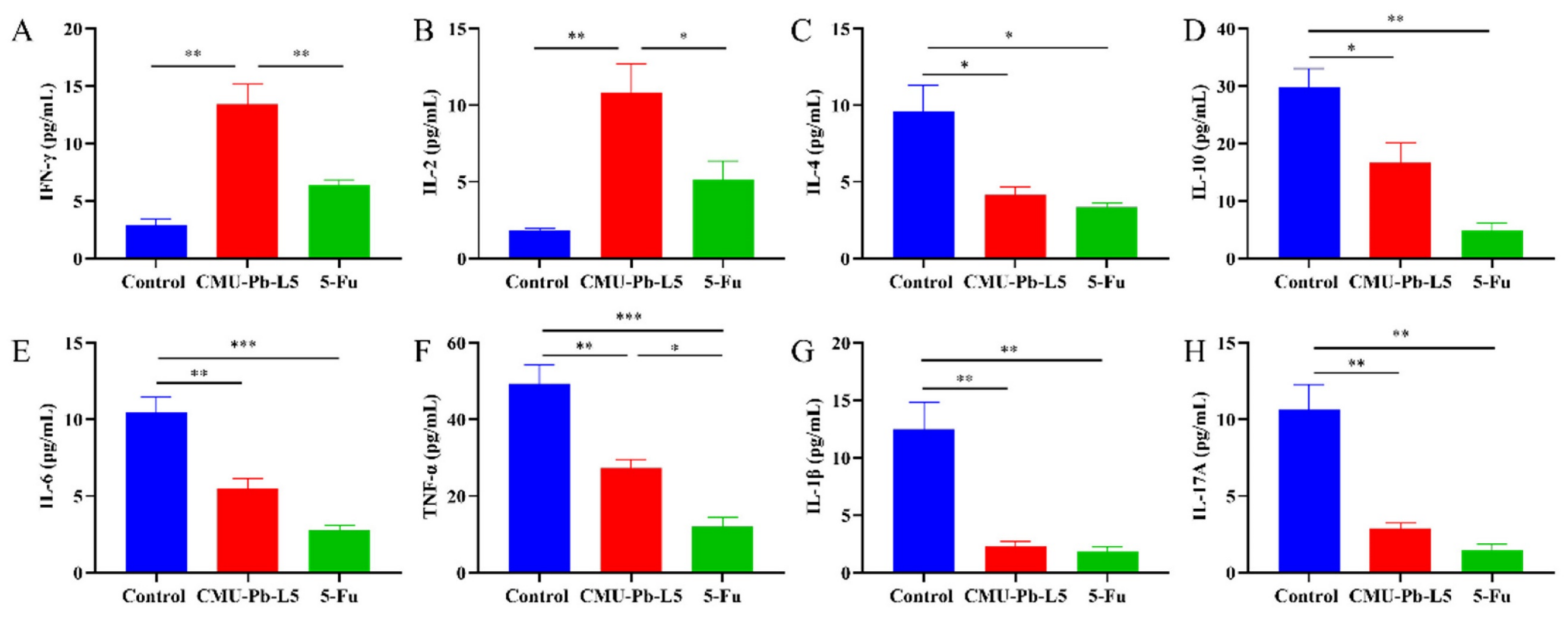
Effects of *L.p* CMU-Pb-L5 on serum immune-related cytokines in mice. The levels of IFN-γ (**A**), IL-2 (**B**), IL-4 (**C**), IL-10 (**D**), IL-6 (**E**), TNF-α (**F**), IL-1β (**G**) and IL-17A (**H**) in mouse serum were analyzed by bio-plex pro mouse cytokine assay kit. *, *P* < 0.05; **, *P* < 0.01; ***, *P* < 0.001.

Interferon γ (IFN-γ) is a cytokine associated with anti-tumor immunity, known for its ability to inhibit tumor cell growth, promote tumor cell apoptosis, and enhance the cytotoxicity of T cells, playing a pivotal role in anti-tumor immunity^31^. IL-2 is crucial for the differentiation of CD4^+^ T cells, promoting the activity of CD8^+^ T cells and NK cells, and exhibiting anti-tumor effects *in vivo*^32^. In our experiment, the levels of IFN-γ and IL-2 in CMU-Pb-L5 group were significantly higher than those in other groups (*P* < 0.01) (Figure 7A, B). Conversely, the tumor microenvironment contains immunosuppressive cytokines that can facilitate cancer cell survival, proliferation, and ultimately tumor development. IL-10 and IL-4 are confirmed immunosuppressive cytokines associated with tumor growth and metastasis^33^. In our study, the levels of IL-4 and IL-10 were significantly decreased in CMU-Pb-L5 group (*P* < 0.05) (Figure 7C, D). Additionally, the levels of inflammatory cytokines IL-6, TNF-α, IL-1β, and IL-17A were significantly reduced in CMU-Pb-L5 group (*P* < 0.05) (Figure 7E-H).

### 3.8 *L.p* CMU-Pb-L5 interfered the apoptosis of tumor tissue in mice

**Figure 8:**
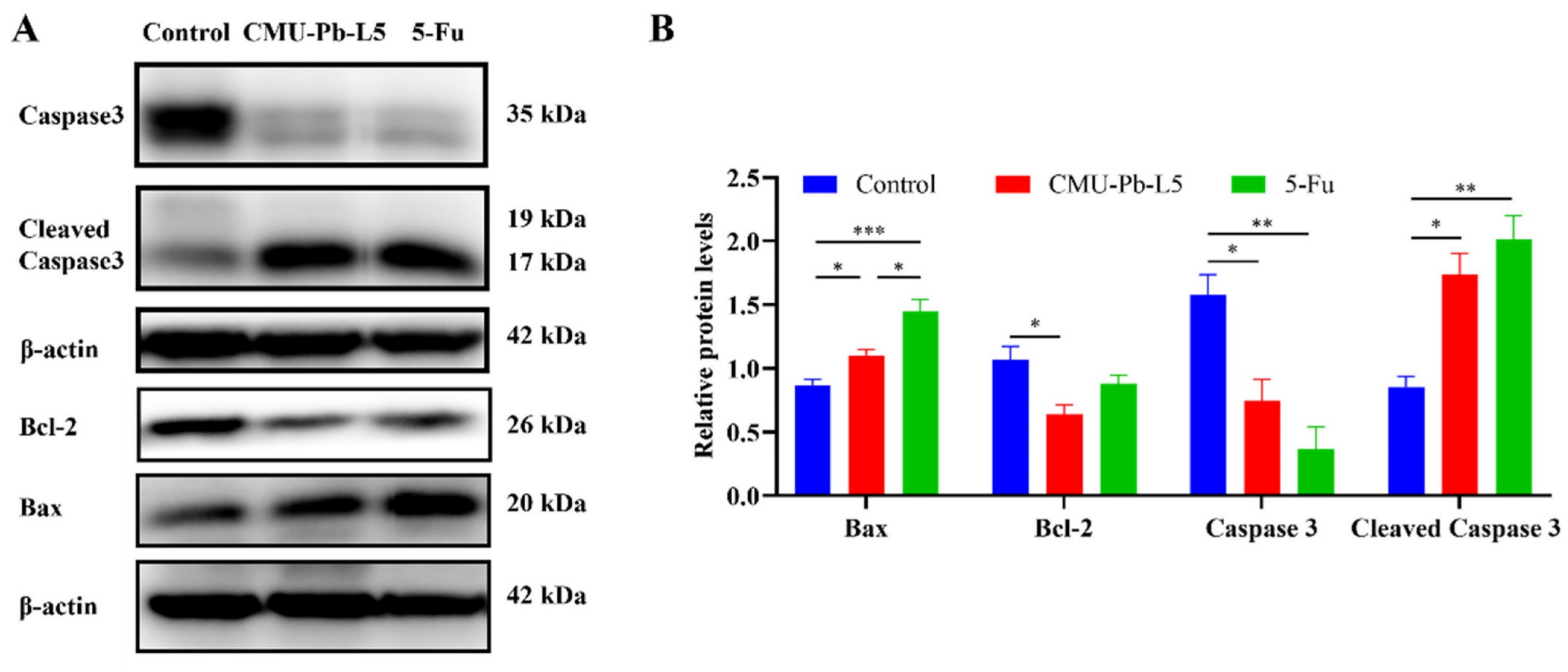
Effects of *L.p* CMU-Pb-L5 on apoptosis-related protein expression. (**A**) The expression of Caspase3, Cleaved Caspase3, Bcl-2, and Bax in mice tumor tissues were detected by Western blot. (**B**) Representative semi-quantification of protein band densities normalized to β-actin from the Western blot analysis. *, *P* < 0.05; **, *P* < 0.01; ***, *P* < 0.001.

We assessed the expression of apoptosis-related proteins in tumor tissues of mice in each group. The levels of pro-apoptotic proteins Bax and Cleaved Caspase3 in tumor tissues were significantly increased following *L.p* CMU-Pb-L5 intervention, while the expression level of the anti-apoptotic protein Bcl-2 was significantly decreased (Figure 8).

## 4. Discussion

*Lactobacillus paracasei* is a common probiotic strain known for its robust intestinal colonization ability, immune enhancement, regulation of intestinal flora, inhibition of pathogenic bacteria, and anti-tumor activity. Previous studies^27^ have confirmed that *L.p* CMU-Pb-L5, isolated from healthy human feces, forms strong biofilms, withstands gastric and intestinal fluids, is safe for ingestion, and significantly reduces pro-inflammatory cytokine expression in mouse colitis models, thereby improving symptoms and pathology. In this study, we further investigated the anticancer activity and related inhibitory effects of *L.p* CMU-Pb-L5 on CRC, aiming to provide a basis for its potential clinical application.

To assess the antitumor activity of *L.p* CMU-Pb-L5, we conducted *in vitro* experiments by co-culturing *L.p* CMU-Pb-L5 with human CRC cell lines SW1116 and RKO, followed by measuring cell proliferation using the CCK-8 method. The results demonstrated that *L.p* CMU-Pb-L5 inhibited CRC cell proliferation in a dose-dependent manner and promoted apoptosis of SW1116 and RKO cells. In an established mouse model of CRC, we observed significant reductions in tumor volume and weight after administration of *L.p* CMU-Pb-L5, indicating its inhibitory effect on tumor growth in mice. Additionally, *L.p* CMU-Pb-L5 effectively reduced the expression of Ki-67 protein in CRC tumor cells, thereby inhibiting tumor proliferation to some extent, consistent with previous studies by Vinícius da Silva Duarte *et al.*^34^.

Polyamines, including putrescine, spermidine, and spermine, are crucial for various cellular processes such as proliferation, differentiation, and DNA synthesis^35^. Disturbances in polyamine homeostasis are closely linked to cancer development, with elevated polyamine levels and ODC1 expression observed in many epithelial tissue-associated cancers like CRC^36^. In CRC, polyamine levels are higher in tumor tissues compared to neighboring tissues and non-cancer patients^37^. These compounds are essential for tumor cell proliferation and also for maintaining intestinal mucosal function^38^. Therefore, consumption of polyamines (inhibiting synthesis, promoting breakdown, and reducing intake) was an effective way to inhibit the growth of CRC^39^.

SMOX oxidizes spermine to spermidine, generating reactive oxygen species that induce oxidative stress and epithelial cell apoptosis, thereby increasing the risk of tumorigenesis^40^. In this study, we found that the expression levels of SMOX and ODC1 proteins in tumor tissues of mice were significantly decreased after *L.p* CMU-Pb-L5 intervention. This indicates that *L.p* CMU-Pb-L5 inhibits polyamine synthesis by suppressing SMOX and ODC1 expression, thereby reducing polyamine levels in tumor tissues and inhibiting tumor growth. Immunohistochemistry also showed reduced SMOX and ODC1 protein levels in mouse tumor tissues after *L.p* CMU-Pb-L5 treatment, consistent with Western blot results, further supporting *L.p* CMU-Pb-L5's role in inhibiting tumor growth by suppressing polyamine synthesis^41^.

The oncogene c-Myc plays a crucial role in polyamine biosynthesis by upregulating various enzymes' activities^42^, ^43^. In this study, c-Myc was highly expressed in tumor tissues of the control group but significantly reduced after *L.p* CMU-Pb-L5 intervention. This suggests that *L.p* CMU-Pb-L5 reduces c-Myc expression, inhibiting ODC1 transcriptional activity, and thus polyamine biosynthesis, ultimately inhibiting tumor growth. Furthermore, serum levels of SPM and SPD were higher in control group but decreased after *L.p* CMU-Pb-L5 intervention. This indicates that *L.p* CMU-Pb-L5 intervention reduces SPM and SPD levels *in vivo*, contributing to tumor growth inhibition. These findings align with previous reports on *Lactobacillus* strains reducing ODC1 expression in CRC models.

The enhancement of T cell infiltration in tumors through the modulation of gut microbiota is a popular area of research. Recently, in a study conducted by Zhang *et al.* revealed that *Lactobacillus paracasei sh2020* stimulation triggered the upregulation of CXCL10 expression in tumors, which in consequence enhanced the recruitment of CD8^+^ T cells, then reduced the tumor burden in CRC mice model^44^. With this in mind, we will further investigate whether CMU-Pb-L5 could affect T cell infiltration in tumors in CRC model mice.

Several studies have demonstrated that *Lactobacillus acidophilus* significantly increases the content of IFN-γ in mice, inhibits angiogenesis, and enhances the activity of natural killer cells, thus exerting an anti-tumor immune effect and inhibiting tumor growth^45^. Additionally, intervention with *L.p* BL23 has been reported to elevate the content of IL-2 in mice. High levels of IL-2 can activate the function of effector T cells and NK cells, contributing to an anti-tumor immune role^46^. Conversely, IL-10 and IL-4, as immunosuppressive cytokines in the tumor microenvironment, have been shown to weaken the anti-tumor immune response and promote immune escape of tumor cells, thereby supporting cancer cell survival and proliferation^47^, ^48^. In our study, we observed a significant increase in IFN-γ and IL-2 levels in mice following *L.p* CMU-Pb-L5 intervention, suggesting that *L.p* CMU-Pb-L5 may regulate the expression of anti-tumor immune-related cytokines to inhibit tumor growth. Moreover, levels of IL-10 and IL-4 decreased significantly in CMU-Pb-L5 group, indicating that *L.p* CMU-Pb-L5 intervention could reduce the expression of immunosuppressive cytokines *in vivo*, thus improving the tumor immune microenvironment and inhibiting tumor growth.

Inflammatory cytokines play crucial roles in tumor development. Desimone *et al.*[49]demonstrated that inflammatory cytokines IL-6, IL-17A, and TNF synergistically activate STAT3 and NF-κB signaling pathways, promoting the occurrence and development of colorectal cancer (CRC). IL-6 and IL-1β are highly expressed in human CRC tissues, playing significant roles in regulating CRC progression and growth. Our study found a significant decrease in IL-6, IL-1β, IL-17A, and TNF-α levels in mice after *L.p* CMU-Pb-L5 intervention, indicating that *L.p* CMU-Pb-L5 intervention could reduce inflammatory cytokine levels *in vivo* and slow CRC tumor growth.

*Lactobacillus* played a significant role in regulating CRC cell proliferation and apoptosis. Co-culture of *L.p* and HT29 cells *in vitro* demonstrated activation of apoptosis-related signaling pathways, upregulation of pro-apoptotic proteins (Bax, Caspase3, and Caspase9), and downregulation of anti-apoptotic protein Bcl-2^50^. Spermine (SPM) and spermidine (SPD) have been shown to inhibit tumor cell apoptosis, promoting tumor occurrence and development. Apoptosis induced by polyamine depletion occurs through the mitochondrial pathway, characterized by mitochondrial membrane potential loss, increased expression of pro-apoptotic protein Bax, and decreased expression of anti-apoptotic proteins Bcl-xl and Bcl-2^51^, ^52^. In our study, after *L.p* CMU-Pb-L5 intervention, SPM and SPD contents in mouse serum were reduced. Considering the relationship between polyamines and apoptosis, we further analyzed the expression of apoptosis-related proteins in mouse tumor tissues in each group. The results showed a significant reduction in the expression level of anti-apoptotic protein Bcl-2 and significant increases in the expression levels of pro-apoptotic protein Bax and Cleaved Caspase3 in mouse tumor tissues following *L.p* CMU-Pb-L5 intervention. These results suggest that *L.p* CMU-Pb-L5 intervention can promote tumor cell apoptosis and inhibit tumor growth, possibly through decreased polyamine content *in vivo*.

## 5. Conclusion

The experimental results of this study demonstrate that *Lactobacillus Paracasei* strain CMU-Pb-L5 (*L.p* CMU-Pb-L5) inhibits tumor cell proliferation in colorectal cancer (CRC) mice, promotes tumor cell apoptosis, reduces polyamine content, and enhances the immune response, thereby suppressing tumor occurrence and progression. Hence, we propose that *L.p* CMU-Pb-L5 holds promising clinical application potential in CRC treatment. However, further investigation is warranted to elucidate which components of *L.p* CMU-Pb-L5 influence polyamine metabolism and subsequently inhibit tumor growth. Future experiments could focus on the combination of *L.p* CMU-Pb-L5 with inhibitors related to polyamine metabolism to elucidate the specific mechanism by which *L.p* CMU-Pb-L5 inhibits CRC growth.
